# Brain-derived neurotrophic factor (BDNF) -TrKB signaling modulates cancer-endothelial cells interaction and affects the outcomes of triple negative breast cancer

**DOI:** 10.1371/journal.pone.0178173

**Published:** 2017-06-12

**Authors:** Yi-Fang Tsai, Ling-Ming Tseng, Chih-Yi Hsu, Muh-Hwa Yang, Jen-Hwey Chiu, Yi-Ming Shyr

**Affiliations:** 1 Comprehensive Breast Health Center & Division of General Surgery, Department of Surgery, Taipei Veterans General Hospital, Taipei, Taiwan, Republic of China; 2 Institute of Clinical Medicine, Institute of Traditional Medicine, School of Medicine, National Yang-Ming University, Taipei, Taiwan, Republic of China; 3 Department of Surgery, School of Medicine, National Yang-Ming University, Taipei, Taiwan, Republic of China; 4 Department of Pathology and Laboratory Medicine, Taipei Veterans General Hospital, Taiwan, Republic of China; 5 School of Medicine, National Yang-Ming University, Taipei, Taiwan, Republic of China; 6 Division of Medical Oncology, Department of Medicine, Taipei Veterans General Hospital, Taipei, Taiwan, Republic of China; 7 Genomic Medicine Research Center, Taipei Veterans General Hospital, Taipei, Taiwan, Republic of China; 8 Division of General Surgery, Department of Surgery, Cheng-Hsin General Hospital, Taipei, Taiwan, Republic of China; 9 Institute of Traditional Medicine, School of Medicine, National Yang-Ming University, Taipei, Taiwan, Republic of China; University of South Alabama Mitchell Cancer Institute, UNITED STATES

## Abstract

**Aims:**

There is good evidence that the tumor microenvironment plays an important role in cancer metastasis and progression. Our previous studies have shown that brain-derived neurotrophic factor (BDNF) participates in the process of metastasis and in the migration of cancer cells. The aim of this study was to investigate the role of BDNF on the tumor cell microenvironment, namely, the cancer cell-endothelial cell interaction of TNBC cells.

**Methods:**

We conducted oligoneucleotide microarray analysis of potential biomarkers that are able to differentiate recurrent TNBC from non-recurrent TNBC. The MDA-MB-231 and human endothelial HUVEC lines were used for this study and our approaches included functional studies, such as migration assay, as well as Western blot and real-time PCR analysis of migration and angiogenic signaling. In addition, we analyzed the survival outcome of TNBC breast cancer patients according to their expression level of BDNF using clinical samples.

**Results:**

The results demonstrated that BDNF was able to bring about autocrinal (MDA-MB-231) and paracrinal (HUVECs) regulation of BDNF-TrkB gene expression and this affected cell migratory activity. The BDNF-induced migratory activity was blocked by inhibitors of ERK, PI3K and TrkB when MDA-MB-231 cells were examined, but only an inhibitor of ERK blocked this activity when HUVEC cells were used. Furthermore, decreased migratory activity was found for △BDNF and △TrkB cell lines. Ingenuity pathway analysis (IPA) of MDA-MB-231 cells showed that BDNF is a key factor that is able to regulate a network made up of metalloproteases and calmodulin. Protein expression levels in a tissue array of tumor slices were found to be correlated with patient prognosis and the results showed that there was significant correlation of TrkB expression, but not of BDNF. expressionwith patient DFS and OS.

**Conclusion:**

Our study demonstrates that up-regulation of the BDNF signaling pathway seems tobe involved in the mechanism associated with early recurrence in triple negative breast cancer cell. In addition, BDNF can function in either an autocrine or a paracrine manner to increase the migration ability of both MDA-MB-231 cells and HUVEC cells. Finally, overexpression of TrkB, but not of BDNF, is significantly associated with a poor survival outcome for TNBC patients.

## Introduction

Research on triple negative breast cancer (TNBC) remains limited area compared to other subtypes of breast cancer and exhibits novel features including heterogeneity and various stemness characteristics. In spite of the availability of the multiple modalities for the treatment of breast cancer that have been developed over the past decades, chemotherapy remains the main treatment for TNBC. This approach does not result in survival levels and outcomes as good as those of other subtypes of breast cancer. Overall, TNBC accounts for approximately 15% of invasive breast cancers [1, 2] and is generally found to be high grade and have a high proliferative index. Recent evidence has suggested that TNBC may be composed of a number of distinct subtypes; these have been identified either by gene expression analysis [3]or using clinical/pathological correlations [4]. This heterogeneity impacts on clinical survival and outcome and differs between the various subtypes [5]. Some subtypes may relapse quickly despite several types of therapy being used, while others may remain as a stable disease after proper adjuvant treatment. Although some promising targets, such as PARP and the genes involved in the DNA damage pathway, have been proposed as therapeutic targets for TNBC treatment, the critical biomarkers that are involved in the process of early metastasis still remains to be investigated in detail.

There has been good evidence to show that the tumor microenvironment plays an important role in cancer metastasis and progression [6–8]. The cell types involved in the interactions between cancer cells and their microenvironment are not limited to stromal cells [9], but other cell types, such as macrophage and endothelial cells, are also involved. For example, Snail, when acetylated, modulates cancer cells to promote the recruitment of macrophages [10]. In addition, the extracellular matrix plays a crucial role in cancer cell growth and how these interact with their adjacent endothelial cells [11]. Recent investigations have suggested that phosphorylation of Src and stromal-derived factor-1 (SDF-1α) are key factors involved in the promotion of bone metastasis [12] and in the tumor progression of estrogen-negative breast cancers [13]. Nonetheless, the role of brain-derived neurotrophic factor (BDNF) in the interaction between breast cancer cells and endothelial cells interaction remains unknown.

Brain-derived neurotrophic factor (BDNF), a member of the “neutrophin” family is widely expressed in mammalian brain. BDNF is well known to support the survival of existing neurons and to encourage the growth and differentiation of new neurons and synapses [14, 15]. Previous research has focused on how BDNF is involved in neural development, neural regeneration, muscle repair, muscle regeneration and muscle differentiation [16–18] BDNF activates various biological functions and this mainly occurs via one cell surface tyrosine kinase receptor, namely tropomyosin receptor kinase B (TrkB). Recent evidence has emphasized the importance of the BDNF/TrkB signaling pathway in the regulation of carcinogenesis and metastasis [19]. Moreover, overexpression of BDNF and TrkB has been demonstrated to act as a predictor of a poor clinical outcome and a worse survival when patients are suffering from human bladder cancer, neuroblastoma and breast carcinoma [20–22].

BDNF/TrkB signaling plays important roles in tumor metastasis and is recognized as a therapeutic target in treatment of breast cancer [22]. While controversy that BDNF predicts adverse pathological and clinical outcome in breast cancer patients remains [23], the precise mechanism by which BDNF is involved in cancer cell signaling, and how BDNF affects the microenvironment of breast cancers, is need to be further elucidated.

Previously, using genomic microarray analysis of paired (non-recurrent and recurrent) resected tumor tissues obtained from breast cancer patients [24], activation of the BDNF pathway has been found to be crucial to the clinical recurrence of TNBC tumors (Table 1). Furthermore, BDNF is known to modulate a number of endothelial functions, such as promoting angiogenic tube formation [25]and increasing vascular endothelial growth factor (VEGF) expression [26]. Accordingly, it was of interest and is important that the role of BDNF in the tumor cell microenvironment is investigated. To try to answer these questions, in this study we targeted the interaction of cancer cell with endothelial cells using a TNBC cell line.

**Table 1:** Up-regulated pathways in recurrent stage II+IIIA triple negative breast cancer.

Pathways	*p*-value
BDNF signaling pathway	3.81E-10
Integrated Breast Cancer Pathway	4.07E-10
EGF EGFR Signaling Pathway	9.46E-10
Cytoplasmic Ribosomal Proteins	8.34E-09
TSH signaling pathway	1.04E-08
Regulation of Actin Cytoskeleton	1.75E-08
Insulin Signaling	7.48E-08
Wnt Signaling Pathway	1.78E-07
Adipogenesis	1.93E-07
TGF beta Signaling Pathway	2.43E-07
Lymphocyte TarBase	2.90E-07
Prostate Cancer	2.95E-07
Androgen receptor signaling pathway	4.69E-07
Muscle cell TarBase	4.72E-07
Focal Adhesion	6.92E-07
Wnt Signaling Pathway and Pluripotency	1.62E-06
Integrated Pancreatic Cancer Pathway	1.68E-06
Neural Crest Differentiation	1.95E-06

BDNF, brain-derived neurotrophic factor

## Materials and methods

### Cell lines and reagents

The human TNBC cell line MDA-MB-231 (ER-low, PR-low and HER2-low) was obtained from the American Type Culture Collection (ATCC, Manassas, VA, USA) and was maintained in F12 MEM (NO.12400-024,Gibco, NY,USA) supplemented with 2 mM L-glutamine, 10% FBS, and penicillin/streptomycin. The cells were cultured at 37°C in a humidified atmosphere containing 5% CO_2_. Human umbilical vein endothelial cells (HUVEC) (BCRC No.H-UV001) were obtained from the Taiwan Medical Cell and Microbial Resources, Food Industry Research Development Institute, Taiwan and were cultured in Medium 200 supplemented with low serum growth supplement (Invitrogen, Carlsbad, CA, USA). Cells at passages from three to ten were used for all experiments. Recombinant human BDNF (PeproTech, Rocky Hill, NJ, USA) used as a positive control and was purchased commercially. Inhibitors of ERK (PD98059, # P215; Sigma Aldrich Co., Saint Louis, USA), PI3K (LY-294,002, # L9908; Sigma Aldrich Co., Saint Louis, USA), and TrkB (GNF5837, # 4559; Tocris Bioscience Co., Bristol, UK)were commercially available. They were pre-treated 1 h before the administration of BDNF in cell cultures.

### Cell growth by trypan blue dye exclusion assay

TNBC MDA-MB-231 cells and human endothelial cell line (HUVECs) were seeded and transferred into a 12-well plates containing low serum medium (1 x 10^4^/well) for 1 day, 2 days and 3 days. Next the cells were washed twice with phosphate-buffered saline (PBS), pH 7.4, and trypsinized using trypsin-ethylenediamine tetraacetic acid containing 0.05% trypsin, 0.53 mmol/L ethylenediamine tetraacetic acid I 4Na (Gibco/Invitrogen, New York, NY). The resuspended cells were then subjected to a trypan blue dye exclusion assay and the positive cells counted using a hemocytometer.

### Cell migration assay

Cell migration assays were performed using a cell culture insert (NO.80209, ibidi, Munich, Germany). In brief, MDA-MB-231 cells or HUVEC cells at a density of 2×10^4^ cells were seeded into a 3.5 cm Petri dish containing an insert and grown overnight and this was followed by low serum (1% FBS) starvation for 24 h. Next the cells were washed with PBS and the inserts removed. Following this, the cells were allowed to continue to grow for another 24 h. At this point the migrating cells were examined under a light microscope and photographed. Finally, the percentage of migratory cells was calculated.

### Western blotting analysis

Cultured cells (MDA-MB-231 or HUVECs)were lysed in a buffer containing 150 mM KCl, 10 mM Tris pH 7.4, 1% Triton X-100, phasphatase inhibitor and protease inhibitors cocktail (Complete Mini; Roche, Mannheim, Germany). The protein concentration of each cell homogenate was measured by the Bradford’s method [27]. In total, 30μgm of each protein sample was loaded onto a gel in order to carry out 10% SDS-PAGE, after which the separated proteins were transferred onto a PVDF membrane (Hybond-C; Amersham Biosciences, NJ, USA). Following this procedure, the membrane was blocked with 5% bovine serum albumin and then a Western blot hybridization was performed using a number of different specific primary antibodies. The individual antibodies used for the Western blotting were against p-eNOS (Ser1177) (Cell Signaling, #9571, MA, USA), eNOS (Cell Signaling, # 5880,MA, USA), TrkB (BD, 610101), BDNF (Genetex, GTX62495, CA, USA), VEGFA (Genetex, GTX 102643, CA, USA), MMP2 (Cell Signaling, #13132,MA, USA), MMP9 (Cell Signaling, #13667,MA, USA), MMP13 (Genetex, GTX100665, CA, USA), RhoA (Cell Signaling #2117,MA, USA), p-Rac1/cdc42 Ser71 (Cell Signaling #2461,MA, USA), Rac1/2/3 (Cell Signaling, #2465,MA, USA), cdc42 (Cell Signaling, #2466,MA, USA) and COX-2(Cayman Chem160112, Michigan, USA).

### Total RNA extraction and reverse transcription-PCR

Total RNA was isolated by a modified single-step guanidinium thiocyanate method [28] (TRI REAGENT, T-9424, Sigma Chem. Co., St. Louis, MO, USA). Complementary DNA (cDNA) was prepared from the total RNA using a First Strand cDNA Synthesis Kit (Invitrogen, CA, USA). The level of *de novo* gene synthesis of each gene in each experimental group was measured by reverse transcriptase-polymerase chain reaction (RT-PCR). The commercially available primers pairs used in this study were TrkB (Forward5’-AGG GCA ACC CGC CCA CGG AA-3’; Reverse5’-GGA TCG GTC TGG GGA AAA GG-3’), BDNF (Forward 5’-TGG CTG ACA CTT TCG AAC AC-3’; Reverse5’-CCT CAT GGA CAT GTT TGC AG-3’); MMP13 (Forward 5’-GAC TTC CCA GGA ATT GGT GA-3’;Reverse5’-TGA CGC GAA CAA TAC GGT TA-3’); MMP9 (Forward 5’-GCA CGA CGT CTT CCA GTA CC-3’; Reverse 5’-GCA CTG CAG GAT GTC ATA GGT-3’); MMP2 (Forward 5’-GCT GGC TGC CTT AGA ACC TTT C-3’; Reverse 5’-GAA CCA TCA CTA TGT GGG CTG AGA-3’); COX2 (Forward 5’-GCT GAG CCA TAC AGC AAA TCC-3’; Reverse 5-’ GGG AGT CGG GCA ATC ATC AG- 3’); VEGFA (Forward 5’-CTT GCC TTG CTG CTC TAC C-3’; Reverse 5’-CAC ACA GGA TGG CTT GAA G-3’); VEGFR2 (Forward 5’-GTG ACC AAC ATG GAG TCG TG-3’; Reverse 5’-CCA GAG ATT CCA TGC CAC TT-3’), and eNOS (Forward 5’-CCC TTC AGT GGC TGG TAC AT-3’; Reverse5’-TAT CCA GGT CCA TGC AGA CA-3’). In order to exclude the possibility that there was contamination of any PCR component, PCR reactions in the absence of the RT product were also carried out (non-template controls, NTCs); these were performed simultaneously during each set of experiments. The RNA transcripts were quantified and the results were expressed as either the relative expression of the mRNA of specific genes or as the Gene/RPLP0 ratio (fold change relative to the control) using the same RNA extract for the controls. All samples were analyzed using triplicate experiments.

### Short hairpin RNA (shRNA) transfection

Short hairpin RNA (shRNA) was used to silence both the BDNF and the TrkB genes and these were obtained from the Academia Sinica. One day after the MDA-MB-231 line had been subcultured, the cells (30–40% confluence) were transfected for 24 h with shRNA against either BDNF or TrkB as well as with anon-silencing control shRNA; the system used for transfection was GenePORTER 2 transfection reagent (Genlantis, San Diego, CA, USA) dissolved in Optimem (Invitrogen) at a final concentration of 80 nM. After the transfections had been carried out the MDA-MB-231 cells were recovered and used for further experiments. After several passages, ΔBDNF and ΔTrkB cell lines were established by puromycin selection. The transfection efficiency was validated by Western blot analysis.

### Subjects

Between Jan. 2001 and Dec. 2010, patients with breast cancer were identified who had been diagnosed with pathological proof at Taipei Veterans General Hospital, Taiwan, ROC. After the approval of the Institutional Review Board (#2013-10-020BC) of this hospital, 95 patients’ records, including date of diagnosis, immunohistochemical (IHC) staining for estrogen receptor (ER) data, progestin receptor (PR) data, IHC staining or fluorescence in situ hybridization (FISH) for HER2 data and subsequent outcome (disease-free survival and overall survival) were retrospectively reviewed; the dataset was obtained from the hospital breast cancer database. Since all clinical data were collected during routine clinical care at the hospital and there had been no direct contact with the patients for the data collection and analysis, written consent by the study subjects was waived by the Institutional Review Board. The mean follow up time was > 60 months. Receptor status was defined to be positive when the ER or PR counts were ≥ 1%. The status was negative when the ER or PR counts were < 1%

### Immunohistochemistry to measure BDNF and TrkB expression

The protein expression levels of BDNF and TrkB in a tissue array containing 95 tumor samples from the archives of the Department of Pathology were measured after immunohistochemical staining for BDNF (Genetex, GTX62495,CA, USA) and TrkB (BD, 610101); the slides were evaluated by an expert in pathology. The degree of positiveness for protein expression was measured in a semi-quantified manner and is expressed as (0), <10%, (1), 11–25%, (2), 26–50%, (3) >50% of the tumor cells examined.

### Statistical analysis

Data are expressed as the mean ± SEM. Differences between time and concentration effect were analyzed by two-way ANOVA. Differences between groups were identified by one-way ANOVA or repeatedly measured one way ANOVA, which was followed by Dunnet’s *post hoc* test. Statistical comparison between two independent groups was determined by the Mann-Whitney U test. A *p* value of <0.05 was considered statistically significant compared to the vehicle group or the no treatment group. All of the above analyses were carried out using GraphPad Prism 5 software.

Disease-free survival (DFS) was defined as the time between initial breast cancer diagnosis and the date of recurrence confirmed by imaging study or by pathological confirmation. Overall survival (OS) was calculated from the time of initial breast cancer diagnosis to the date of death or last consultation. The Kaplan–Meier method was used to estimate the cumulative incidence of DFS and OS and Log-rank (Mantel-Cox) Test were used for comparisons (GraphPad Prism 5).

## Results

The genomic microarray analysis of the resected tumor tissues obtained from breast cancer patients (GEO, https://www.ncbi.nlm.nih.gov/geo/query/acc.cgi?acc=GSE95700) (GSE95700) (S1 File) identified a number of possible critical signaling pathway differences between the recurrent and non-recurrent TNBC (S1 Fig). A heat map analysis of the samples (S2 Fig) revealed that the BDNF signaling pathway was a significant predictor of recurrence. (Table 1)

**Fig 1:**
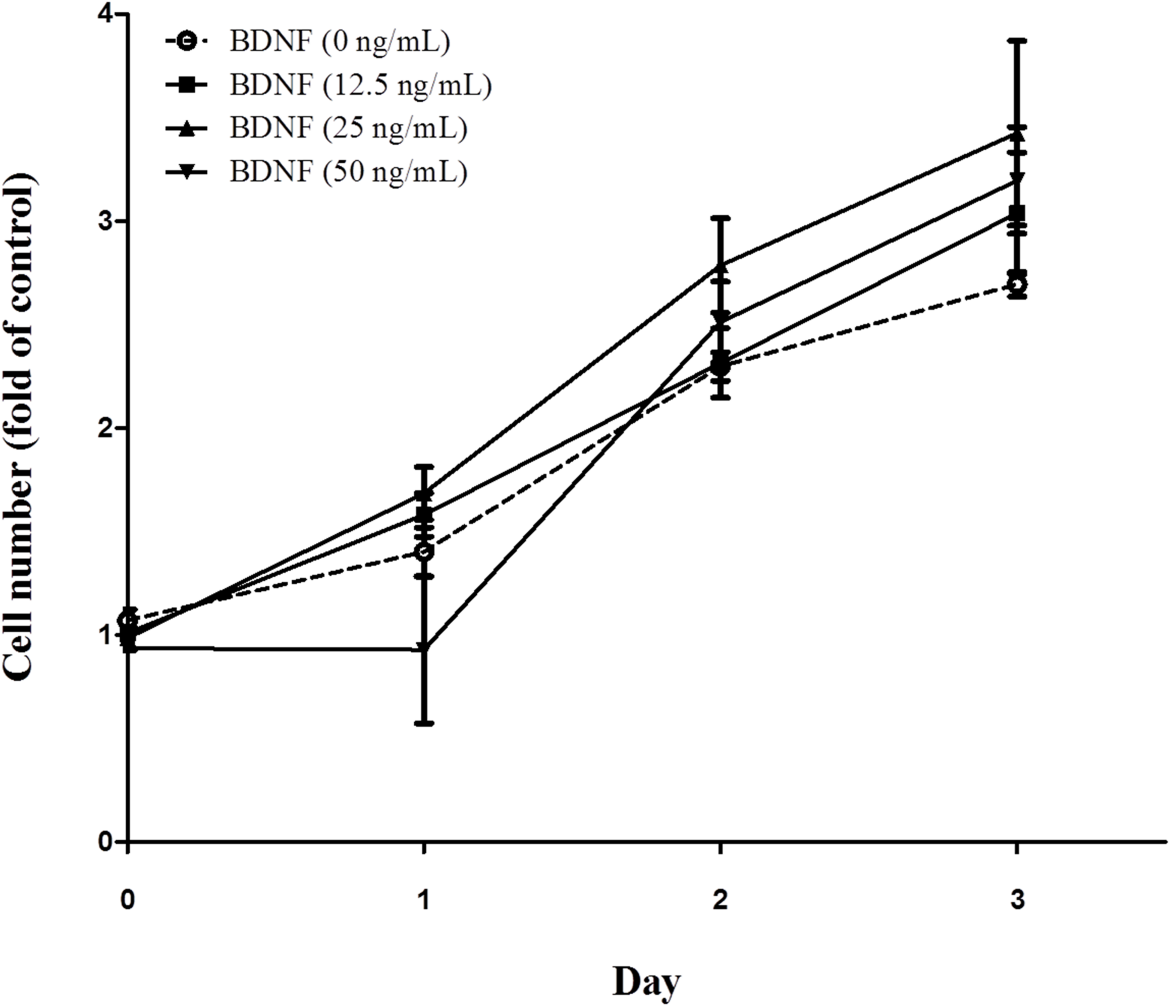
Effect of brain-derived neurotrophic factor (BDNF) on the MDA-MB-231 cell line. MDA-MB-231 cells (1 x 10^4^/well)were seeded and transferred into a 12-well plates containing low serum medium, followed by treatment with BDNF (0-, 12,5-, 25-, and 50 ng/mL)for 1 day, 2 days and 3 days. Then the cells were trypsinized and subjected to a trypan blue dye exclusion assay as described in Methods. (Two way ANOVA, *p*>0.05, n = 6).

Using an *in vitro* system, we found that MDA-MB-231, but not other breast cancer cell lines, secreted BDNF into the culture medium (supplementary reference 4). However, BDNF was not able to stimulate MDA-MB-231 cell proliferation (Fig 1) (Two way ANOVA, *p*>0.05, n = 6)

**Fig 2:**
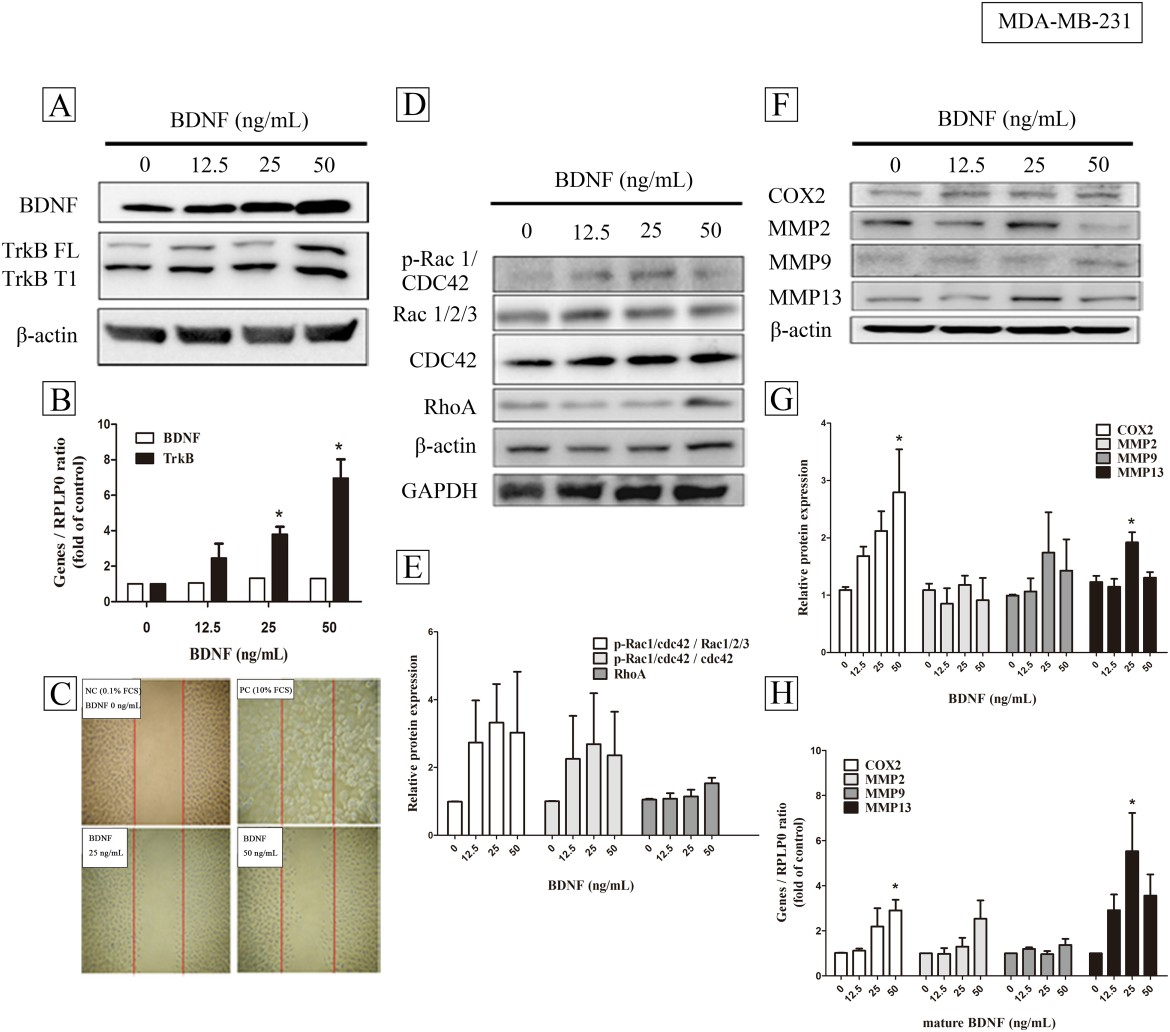
The effects of brain-derived neurotrophic factor (BDNF) on autoregulation, migration and migration related proteins signaling in MDA-MB-231 cells. MDA-MB-231 (4 X10^5^ / well) cells cultured with low serum medium were treated with BDNF (0-, 12,5-, 25-, and 50 ng/mL) for 24 h or 6 h, and analyzed with Western blot (A) or real-time PCR (B), respectively. TkrB FL and TrkB T1 indicated full length and T1 domain of TrkB, respectively. Cells at a density of 2×10^4^ cells were seeded into a 3.5 cm Petri dish containing an insert for migration assay (C) as described in Methods. Protein expressions of Rac, CDC42, and Rho (D) were quantified (E) and gene expression of COX2, MMP2, MMP9, and MMP13 were analyzed with Western blot (F,G) and real-time PCR (H). Data were expressed as mean + SEM. *, *p*< 0.05 compared to vehicle group by one way ANOVA, n = 4–5).

Treatment with BDNF was found to increase the expression of TrkB, but did not affect gene expression of BDNF, either in protein level (Fig 2A) or mRNA level(Fig 2B, one way ANOVA, *p* = 0.0004, n = 4). In addition, treatment of the MDA-MB-231 cell line with BDNF also increased cell migration (Fig 2C) with migrated area (mm^2^) being 0.005±0.001, 0.0884± 0.023, and 0.1087± 0.015 in BDNF 0 -, 25-, and 50 ng/mL treatment, respectively. (one way ANOVA, *p* = 0.001, n = 9). In parallel, there was also a trend of increased p-Rac, CDC42, and Rho protein (Fig 2D and 2E) (one way ANOVA, *p*>0.05, n = 4) and a significantly increased gene expression of COX2 and MMP13, but not MMP2 and MMP9, either in protein level (Fig 2F and 2G) (one way ANOVA, *p*< 0.05, n = 5) or mRNA level(Fig 2H) (one way ANOVA, *p*< 0.05, n = 4).

**Fig 3:**
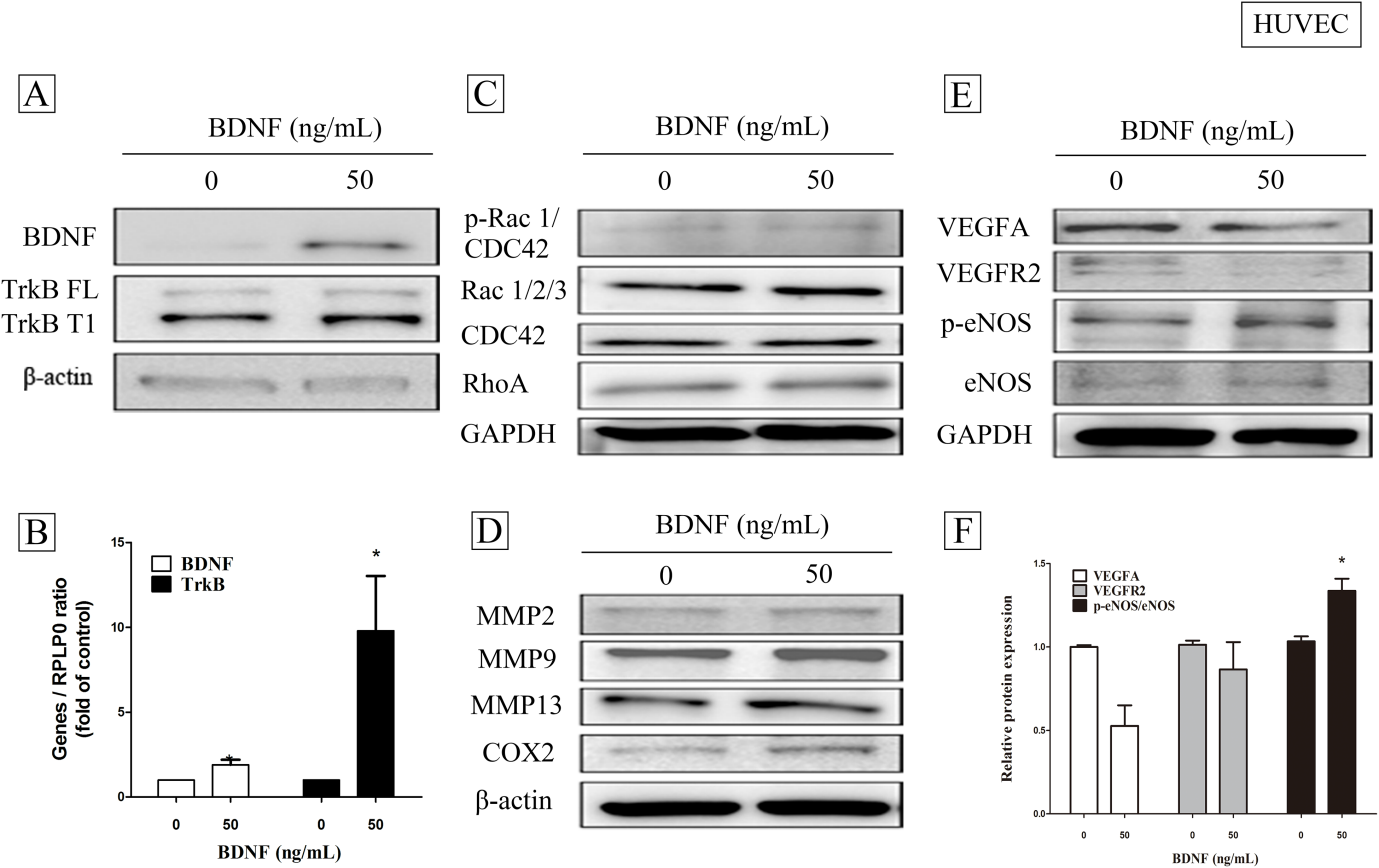
The effects of brain-derived neurotrophic factor (BDNF) on autoregulation and the expression of angiogenesis- related signaling proteins in HUVEC cells. HUVEC cells (4 X10^5^ / well) cultured with low serum medium were treated with BDNF (0-, and 50 ng/mL) for 24 h or 6 h, and analyzed with Western blot (A) or real-time PCR (B), respectively. Protein expressions of Rac, CDC42, and Rho (C) and metastasis-related proteins such as COX2, MMP2, MMP9, and MMP13 (D) were analyzed with Western blot. The angiogenic factors such as VEGFA, BEGFR2, and eNOS were analyzed with Western blot (E) and real-time PCR (F), respectively. Data were expressed as mean + SEM. *, *p*< 0.05 compared to vehicle group by Mann-Whitney U test, n = 4).

In addition to the above findings, BDNF also brought about an increased in HUVEC cells of not only BDNF protein (Fig 3A), but also of BDNF/TrkB mRNA level (Fig 3B) (Mann-Whitney U test, *p* = 0.0294, n = 4). Although there were no increased p-Rac, CDC42, and Rho protein (Fig 3C), nor MMP2, MMP9, MMP13, and COX2 expression (Fig 3D), BDNF increased the activation of eNOS (Fig 3E) and significantly up-regulated eNOS gene expression (Fig 3F) (Mann-Whitney U test, *p* = 0.0286, n = 4), but not angiogenic factors such as VEGFA and VEGFR2 expression.

**Fig 4:**
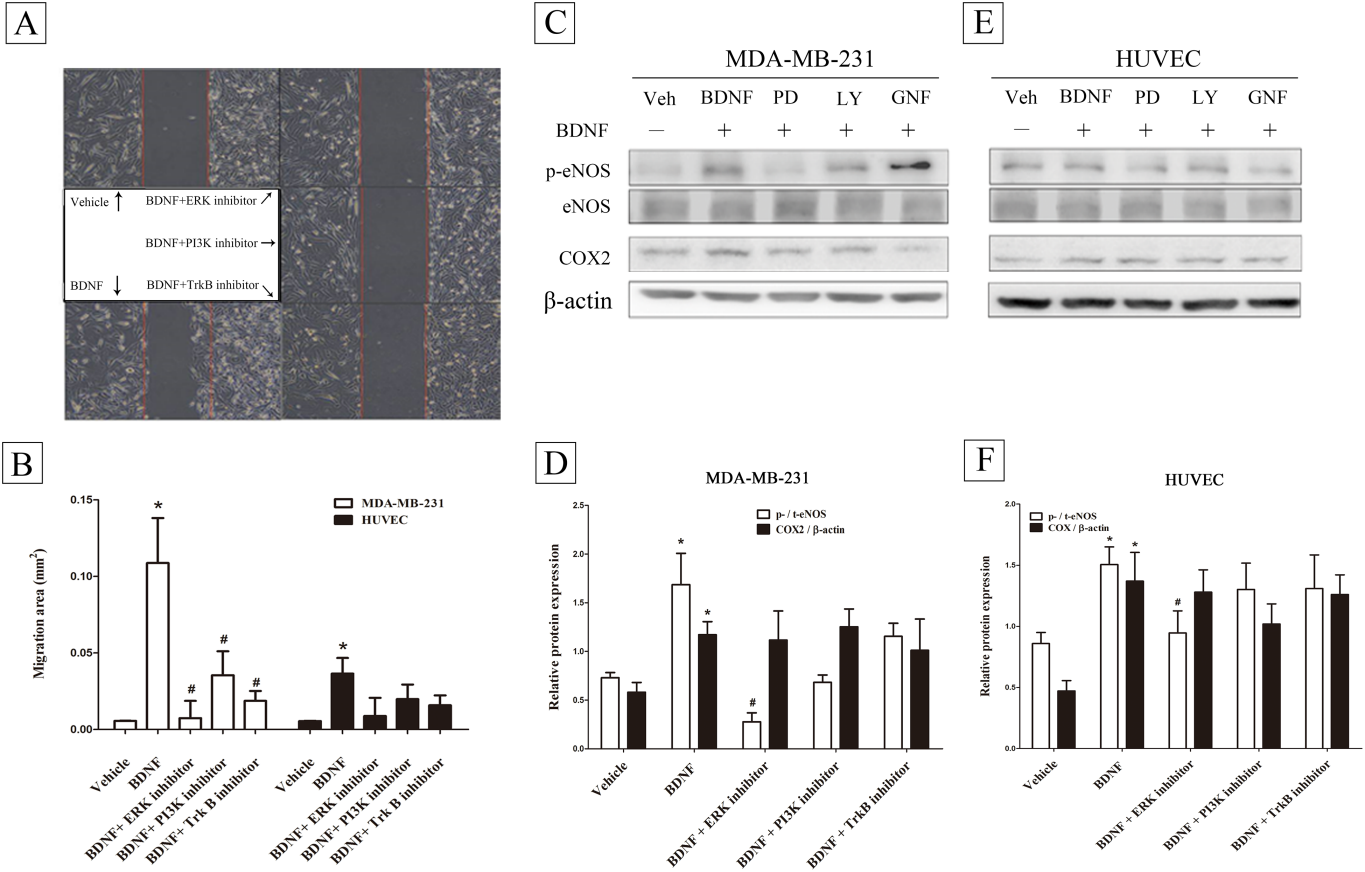
Effects of different inhibitors on brain-derived neurotrophic factor (BDNF) induced migratory activity in the MDA-MB-231 and HUVEC cell lines. MDA-MB-231 cells (2×10^4^) were seeded into a 3.5 cm Petri dish containing an insert for migration assay (A). Inhibitors of ERK, PI3K, and TrkB were pretreated1h before administration of BDNF (50 ng/mL) for 24 h and migratory area (mm^2^) were measured (B) (repeatedly measured one way ANOVA, n = 9)as described in Methods. After with or without pretreatment of inhibitors of ERK, PI3K, and TrkB, BDNF (50 ng/mL)-induced eNOS and COX2 expression in MDA-MB-231 cells (C, D) and HUVEC line (E, F). Data were expressed as mean ± SEM. *, *p*< 0.05 compared to vehicle group by Mann-Whitney U test, n = 5–9).

The above results suggest that BDNF brings about autocrinal and paracrinal regulation of BDNF gene expression. In this context, the BDNF-induced migratory activity (Fig 4A) could be blocked in MDA-MB-231 cell line by treatment with inhibitors of ERK, PI3K and TrkB (Fig 4B)(repeatedly measured one way ANOVA, *p* = 0.0023, n = 9). By way of contrast, the BDNF-induced migratory activity could not be blocked in HUVEC cell line when the cells were treated with ERK, PI3K and TrkB inhibitors. (Fig 4B)(repeatedly measured one way ANOVA, *p>0*.*05*, n = 9). By Western blot analysis on MDA-MB-231 cells (Fig 4C), BDNF-induced p-NOS expression, but not COX2, was blocked by treatment of ERK inhibitor (Fig 4D)(Mann-Whitney U test, *p* = 0.002, n = 4–10). Similarly, Such effects were also observed in HUVEC cells (Fig 4E and 4F) ((Mann-Whitney U test, *p* = 0.0029, n = 5–9). There was no effect when cells were treated with PI3K and TrkB inhibitors.

**Fig 5:**
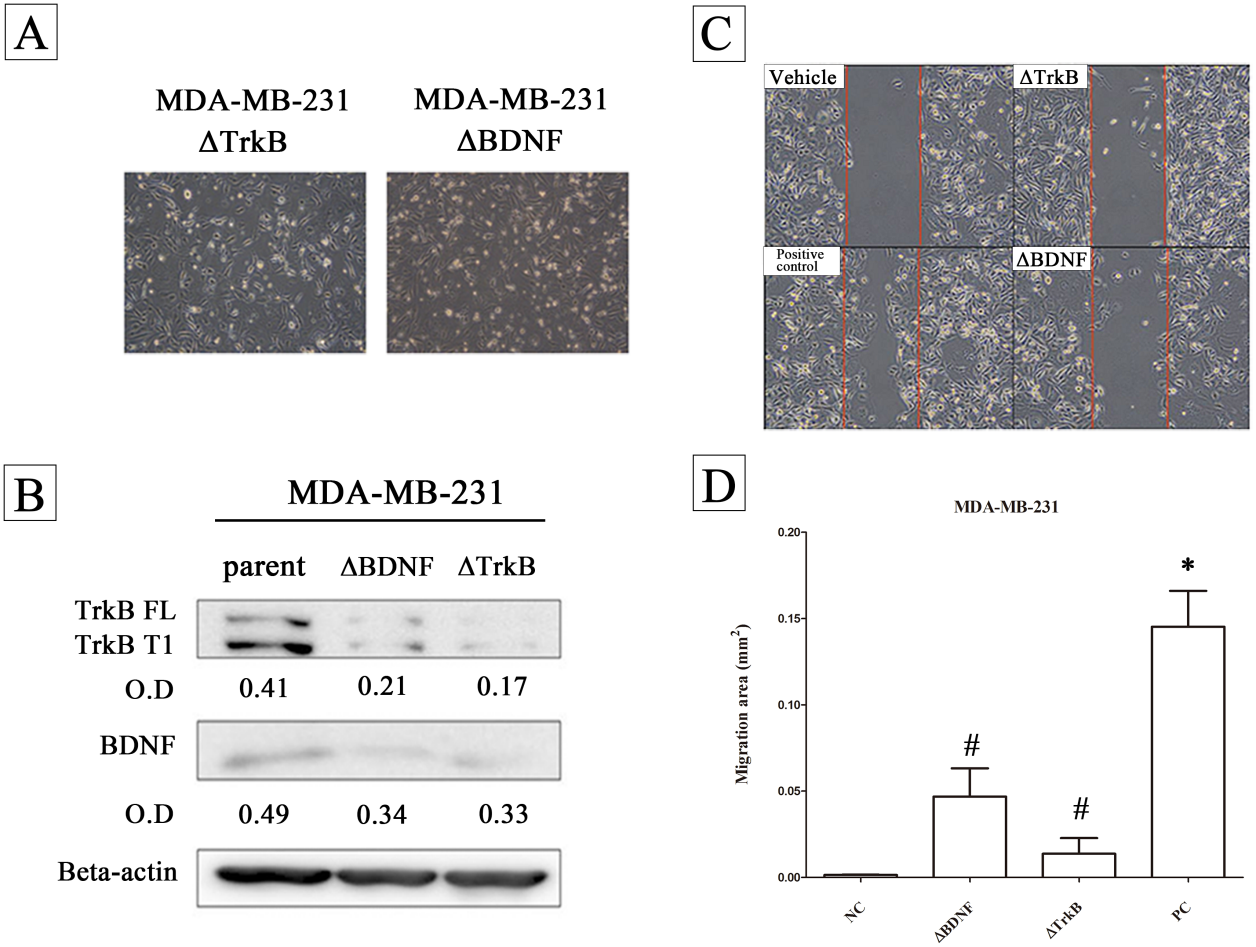
The effects of brain-derived neurotrophic factor (BDNF) and TrkB on the migratory activity of the MDA-MB-231 line. The cell morphology is presented (A) and the protein expression of BDNF and TrkB (B) were validated in the parent, the △BDNF cell line and the △TrkB cell line. MDA-MB-231 parent cells or the △BDNF and the △TrkB cell lines (2×10^4^) were seeded into a 3.5 cm Petri dish containing an insert for migration assay (C). The migratory area was quantified (D). Data were expressed as mean ± SEM. *, *p*< 0.05 compared to vehicle group; #, *p*< 0.01 compared to positive control group by Repeatedly measured one way ANOVA, n = 5).

It should be noted that the BDNF-induced migratory activity in the MDA-MB-231 line was found to be decreased when the parent cells were either *BDNF* knock down (△BDNF) or *NTRK2* (TrkB) knock down (△TrkB) (Fig 5A and 5B). In these cell lines there was also a decrease in migratory activity (Fig 5C and 5D) (Repeatedly measured one way ANOVA, n = 5).

**Fig 6:**
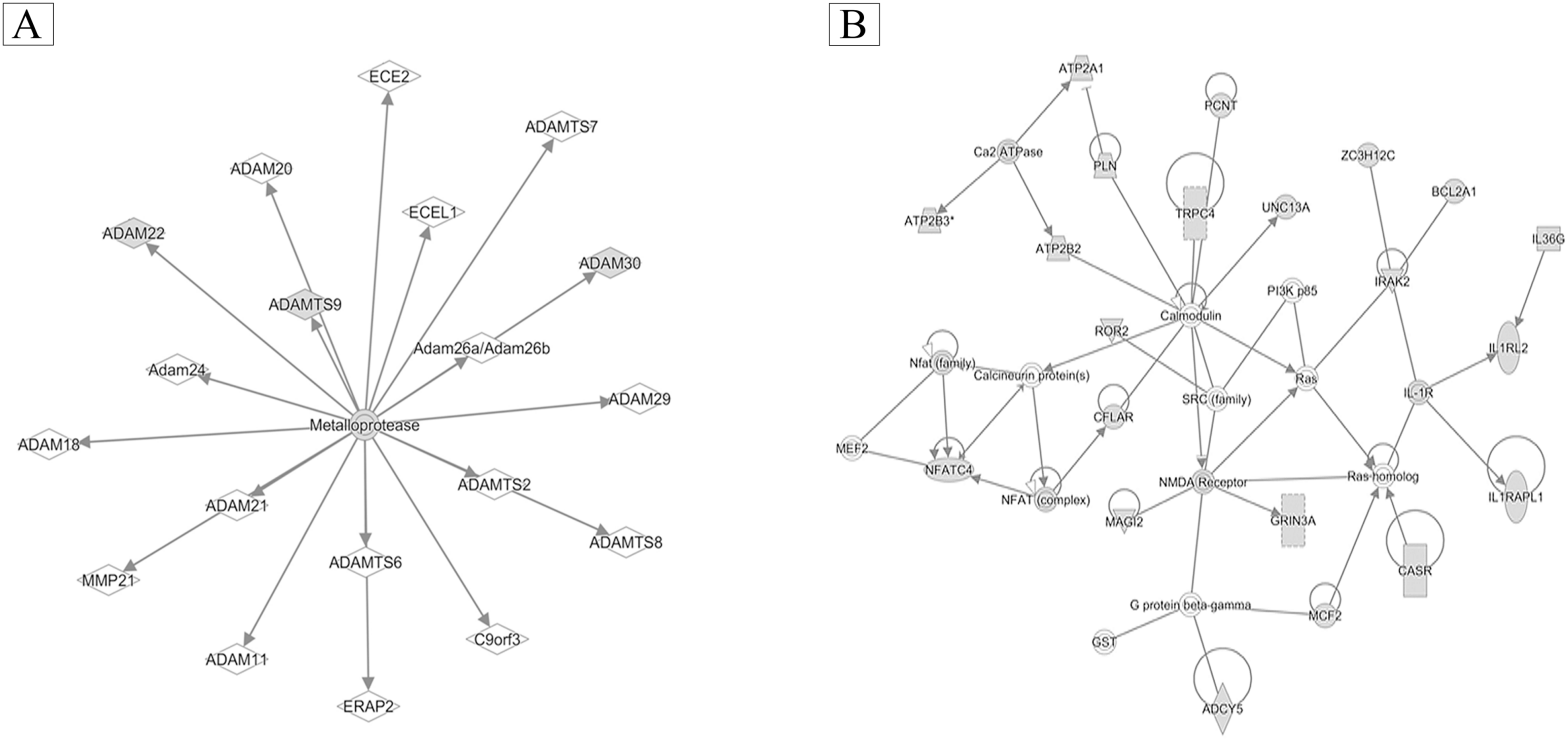
Ingenuity pathway analysis (IPA) of gene expression levels in MDA-MB-231 cells. MDA-MB-231 cells (4 X10^5^ / well) cultured with low serum medium were treated with brain-derived neurotrophic factor (BDNF) (50 μg/mL), followed by RNA extraction and analysis of gene expression by oligonucleotide microarray assay. Then, the possible BDNF-related pathway was analyzed using Ingenuity pathway analysis (IPA). The metalloprotease network (A) and the calmodulin (B) network were modulated by BDNF treatment.

To identify the possible down-stream signaling systems involved in the cross-talk within the BDNF-TrkB pathway, BDNF was administrated to MDA-MB-231 cells, and this was followed by RNA extraction and an examination of mRNA expression using an oligonucleotide microarray assay. Ingenuity pathway analysis (IPA) showed that BDNF was a key factor and was involved in regulating networks of metalloproteases in MDA-MB-231 cells (Fig 6A) together with calmodulin (Fig 6B).

**Fig 7:**
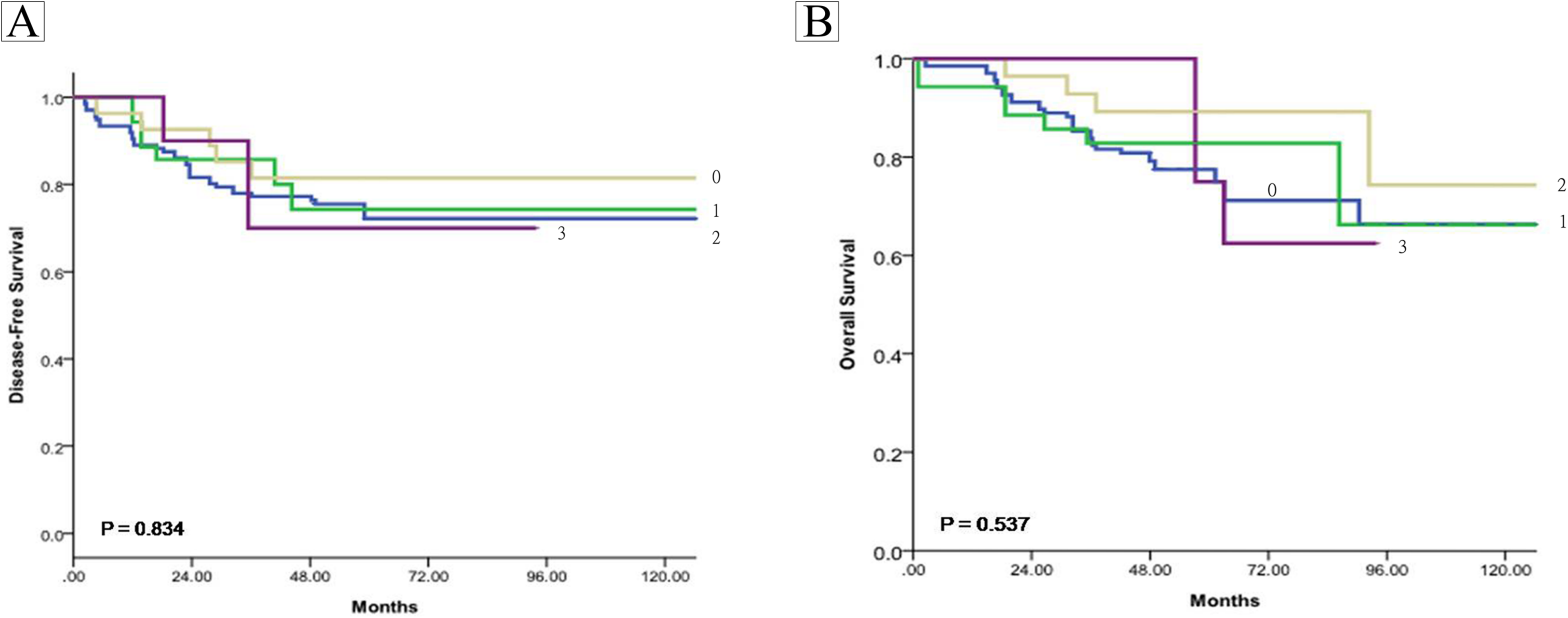
Correlation with their prognosis of brain-derived neurotrophic factor (BDNF) expression levels using a tissue array of tumor slices from patients. The protein expression levels of BDNF in a tissue array were measured after immunohistochemical staining. The degree of positiveness for protein expression was measured in a semi-quantified manner and is expressed as (0), <10%, (1), 11–25%, (2), 26–50%, (3) >50% of the tumor cells examined. Disease-free survival (DFS) and Overall survival (OS) were defined in Methods. The Kaplan–Meier method was used to estimate the cumulative incidence of DFS and OS and Log-rank (Mantel-Cox) Test were used for comparisons.

**Fig 8:**
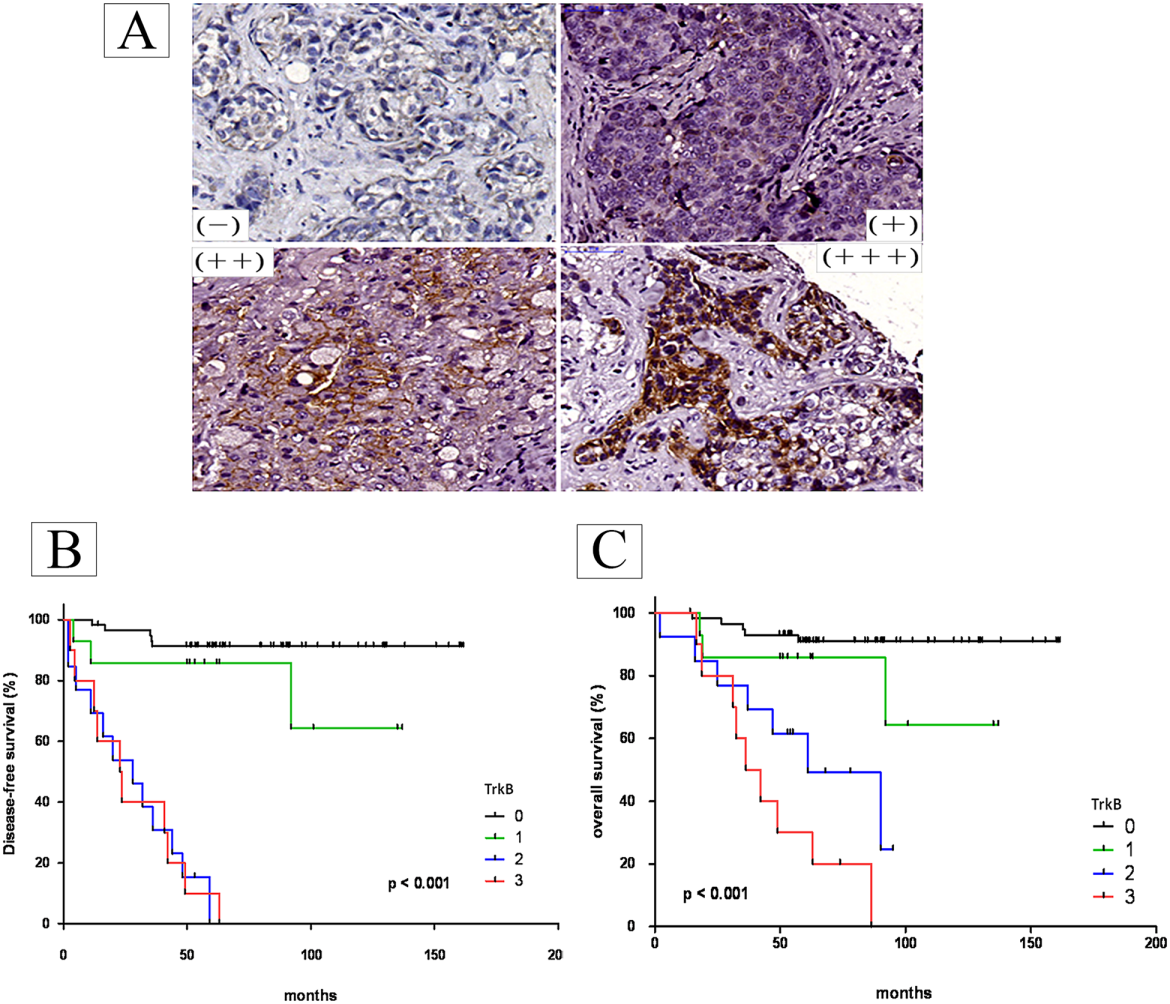
Correlation with their prognosis of TrkB expression levels using a tissue array of tumor slices from patients. The protein expression levels of TrkB in a tissue array were measured after immunohistochemical staining (A). The degree of positiveness for protein expression was measured in a semi-quantified manner and is expressed as (0), <10%, (1), 11–25%, (2), 26–50%, (3) >50% of the tumor cells examined. Disease-free survival (DFS, B) and Overall survival (OS, C) were defined in Methods. The Kaplan–Meier method was used to estimate the cumulative incidence of DFS and OS and Log-rank (Mantel-Cox) Test were used for comparisons.

Finally, we correlated with their prognosis, protein expression levels in a tissue array made up of tumor slices from patients; this was done relative to disease-free survival (DFS) and overall survival (OS). The results showed that there was no significant relationship between BDNA expression and the patients’ DFS (Fig 7A) or OS (Fig 7B). However, by way of contrast, there was a statistically significant relationship between TrkB expression (Fig 8A) and the patients’ DFS (Fig 8B) and OS (Fig 8C).

## Discussion

Cancer metastasis is a process that requires interactions between cancer cells and the tumor microenvironment. In this article, the modulatory effects of BDNF on the interaction between cancer cells (MDA-MB-231) and endothelial cells (HUVEC) was investigated. Previously, we have measured the BDNF level in the cultured media of different breast cancer cell lines (MCF-7, SK-BR-3, MDA-MB-231, MDA-MB-468) and the results showed that only MDA-MB-231 cells, but not other cell types, secreted BDNF into the culture media in a time-dependent manner (S3 Fig). To our knowledge, we are the first to investigate the role of BDNF in cancer cell-endothelial cell interactionsusing breast cancer cells.

Although BDNF is well known to be involved in neural development, regeneration and differentiation [16, 17], several lines of evidence have recently suggested that BDNF and its negative regulatory microRNA may play important roles in cell proliferation and in the metastasis of breast cancer [22, 29]and non-small lung cancer [30]. Moreover, BDNF has been shown to promote non-small lung cancer proliferation in an autocrine manner and metastasis of neuroblastoma through STAT3 and PI3K and MAPKsingalling pathway, respectively [21, 31].

In this study, we demonstrate that BDNF affects not only MDA-MB-231 itself in an autocrine manner, but also affects HUVEC cells in a paracrine manner. These results are in agreetment with previous investigations that have described the role of BDNF in the regulation of cell survival/growth in breast cancer [29, 32], in neuroblastoma and in myeloma, either in an autocrinal manner or in a paracrinal manner [33, 34].

The interactions of cancer cells with their microenvironment are an essential feature of tumor progession and metastasis. The cell types involved in such interactions are not necessarily stromal cells [9], but may include macrophages [10], endothelial cells [11], and T cells [35]. Many factors are well known to be related to the metastatic or invasive potential of human cancer cells. For examples, nitric oxide promotes the invasive activity in lung cancer cells [36]; and changes energy metabolism (increased glycolysis), which affects the tumor-endothelial interaction of bladder carcinoma [37]. Recent evidence has suggested that calmodulin plays a crucial role in the modulation of cancer metastasis during gastric cancer [38]. In addition, the matrix metalloproteases are known to be involved in tumor metastasis [39]. Our results obtained using ingenuity pathway analysis demonstrate that there is linkage between calmodulin expression, expression of MMPs and BDNF expression and it should be noted that this is consistent with above literature. It is also important to note that BDNF and VEGF/VEGFR play different roles in the multi-step process that makes up cancer metastasis [40]. The former promotes the transendothelial migration of cancer cells into vessels and the cells' survival in the circulatory system, while the latter enhances subsequent vascular proliferation at the colonization site. Our results have shown that BDNF increased eNOS, but not VEGF/VEGFR2, expression, and this supports the above hypothesis [41].

In terms of the protein's molecular and clinical roles, BDNF has been reported to be a target marker for predicting clinical outcomes in breast cancers [23, 32]. Nevertheless, controversy still exists as to the exact role of BDNF in tumor suppression and promotion [42]. Our clinical dataset shows that there is a good correlation between TrkB expression and patients’ outcomes, but not BDNF expression and patients’ outcomes, in terms of disease-free survival and overall survival. We attribute the discrepancy in these results to the characteristics of BDNF, which can be secreted outside cells and thus is able to affect the adjacent microenvironment. This may make the quantification of cellular BDNF expression unreliable. However, there is a need for more evidence in order to clarify the exact role of BDNF when predicting patient prognosis.

In summary, we have demonstrated that BDNF-TrkB signaling plays an important role in the interaction between cancer cells (MDA-MB-231) and endothelial cells (HUVEC) and that TrkB expression in tumor cells is correlated with a poor prognosis of patients with TNBC subype cancers.

## Supporting information

S1 File Gene expression omnibus. The data includes the name of the repository (https://www.ncbi.nlm.nih.gov/geo/query/acc.cgi?acc=GSE95700) and the relevant accession numbers (GSM list).
(PDF)

S1 Fig Experimental design. The genomic microarray analysis of the resected tumor tissues obtained from breast cancer patients identified a number of possible critical signaling pathway differences between the recurrent and non-recurrent TNBC.
(TIF)

S2 Fig Heat map analysis. Heat map analysis showed the differentially expressed genes between non-recurrent and recurrent triple negative breast cancer (TNBC). Yellow box contained up-regulated genes in recurrent TNBC compared to non-recurrent tumors.
(TIF)

S3 Fig BDNF level in the cultured media of different breast cancer cell lines. The BDNF level in the cultured media of different breast cancer cell lines (MCF-7, SK-BR-3, MDA-MB-231, MDA-MB-468) were measured and the results showed that only MDA-MB-231 cells, but not other cell types, secreted BDNF into the culture media in a time-dependent manner.
(TIF)
