# Supplementary figures and images for: Brain-derived neurotrophic factor (BDNF) -TrKB signaling modulates cancer-endothelial cells interaction and affects the outcomes of triple negative breast cancer
Source: PLoS One. 2017 Jun 12;12(6):e0178173. doi: 10.1371/journal.pone.0178173 (PMC5467823; doi:10.1371/journal.pone.0178173)

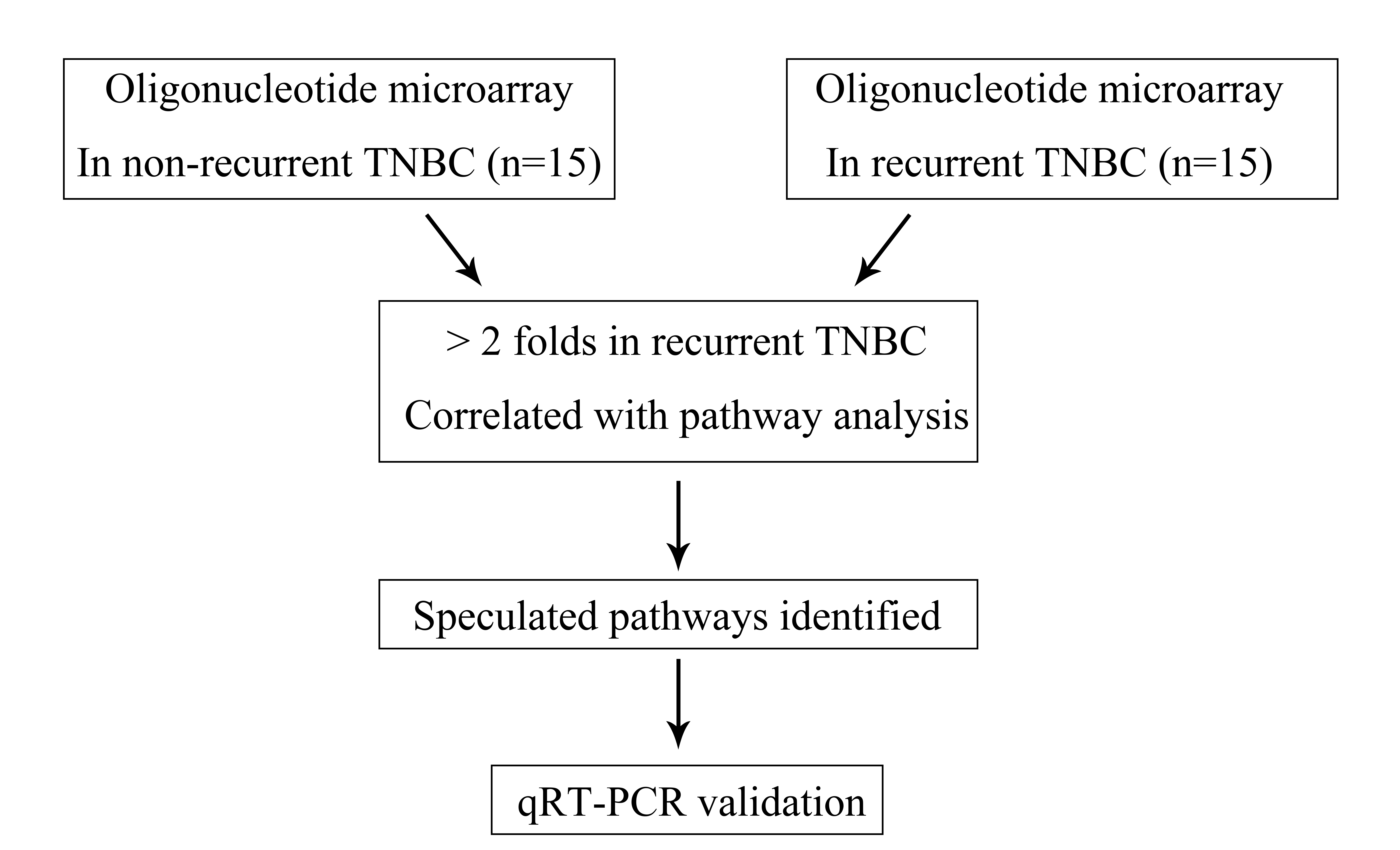

Supplement: S1 Fig — The genomic microarray analysis of the resected tumor tissues obtained from breast cancer patients identified a number of possible critical signaling pathway differences between the recurrent and non-recurrent TNBC. (TIF) [file pone.0178173.s002.tif]

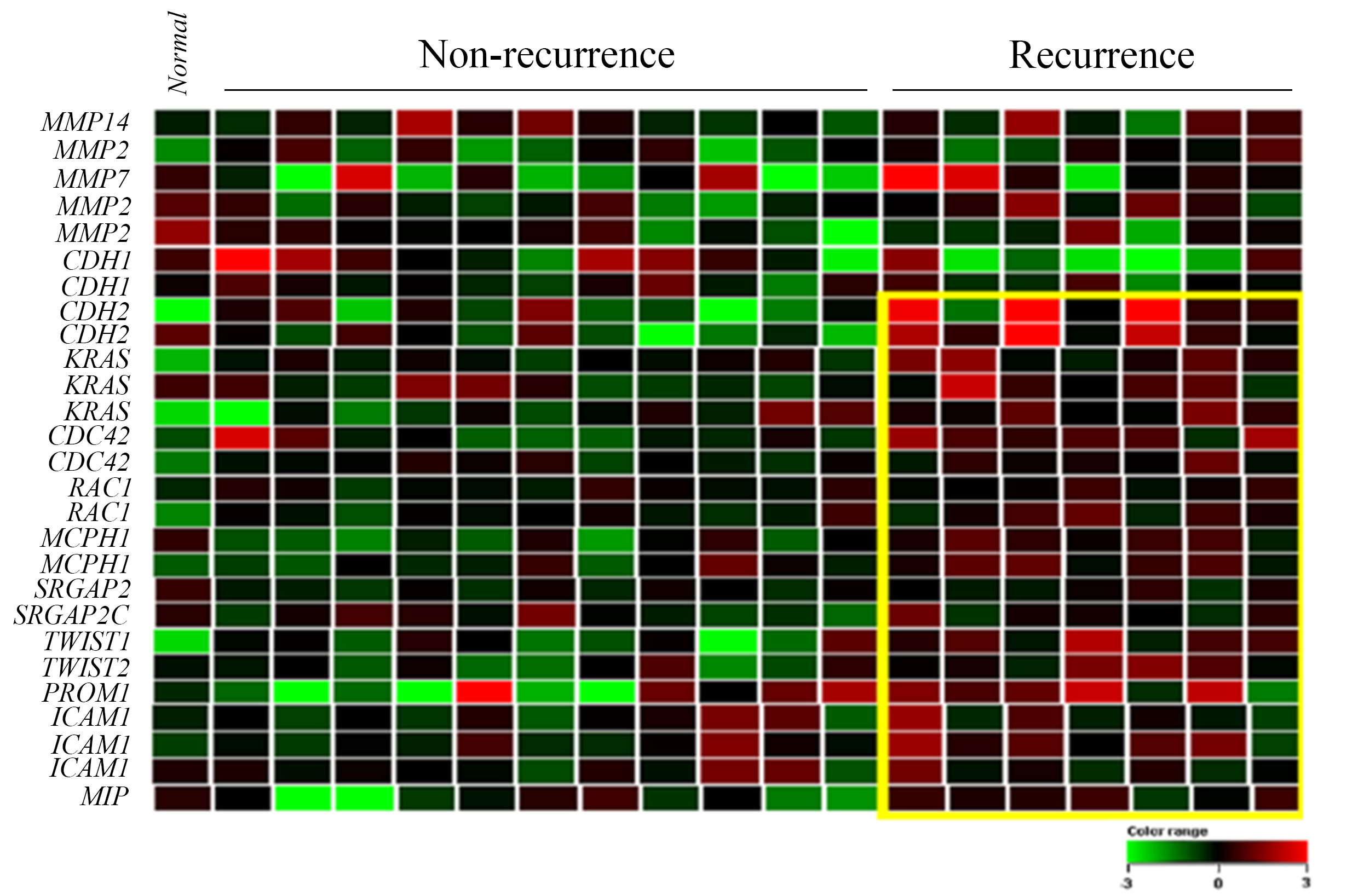

Supplement: S2 Fig — Heat map analysis showed the differentially expressed genes between non-recurrent and recurrent triple negative breast cancer (TNBC). Yellow box contained up-regulated genes in recurrent TNBC compared to non-recurrent tumors. (TIF) [file pone.0178173.s003.tif]
